# Ultrasound-based measurement of optic nerve sheath to evaluate increased intracranial pressure on patients in emergency department

**DOI:** 10.3389/fneur.2025.1539213

**Published:** 2025-02-13

**Authors:** Hai-dan Jiang, Hua-guo Shao, Lin Pan, Hui Li

**Affiliations:** ^1^Department of Ultrasound Imaging, The First Affiliated Hospital of Wenzhou Medical University, Wenzhou, China; ^2^Institute of Hepatology and Epidemiology, Hangzhou Xixi Hospital Affiliated to Zhejiang Chinese Medical University, Hangzhou, China; ^3^Department of Ultrasound, Hangzhou Xixi Hospital Affiliated to Zhejiang Chinese Medical University, Hangzhou, China

**Keywords:** emergency department, intracranial pressure, optic nerve sheath, ultrasound, diagnostics

## Abstract

**Background and aims:**

Recent studies reported that non-invasive imaging techniques for measuring optic nerve sheath diameter (ONSD) offer a novel diagnostic approach for intracranial pressure (ICP) elevation. However, whether ultrasound-measured ONSD sensitively evaluate the real-time ICP is unknown. This study explores the ability of different measurement modalities to predict ICP elevation, aiming to provide a superior non-invasive ICP monitoring technique for clinical practice.

**Methods:**

Optic nerve sheath (ONS) measurement using three modalities and ICP of 104 patients collected from January 2018 to May 2021 were analysis by correlation analysis and receiver operating characteristic curve analysis.

**Results:**

Significant correlations were observed between ICP and ONS measurement using three modalities (*p* < 0.001). According to the ROC analysis, if ONS long diameter > 5.55 mm, ONS vertical diameter > 5.75 mm, left ONS area > 25.05 mm^2^ or right ONS area > 25.73 mm^2^, the patient was considered to have elevated ICP.

**Conclusion:**

Ultrasonic transverse scanning, longitudinal scanning, and area-based measurement of the retrobulbar ONS are excellent screening tools for the diagnosis of ICP. These three methods exhibited nearly identical levels of correlation, sensitivity, and specificity. All three measurement approaches demonstrated capabilities in diagnosing elevated ICP.

## Introduction

High intracranial pressure (ICP) represents a severe pathological condition that arises from various etiologies, encompassing craniocerebral injury, intracranial space-occupying lesions, cerebrospinal fluid circulation disorders, idiopathic causes, or major venous sinus occlusion (1, 2). While invasive ICP measurement through intracranial devices or lumbar puncture (LP) is feasible, the lack of specialized neurosurgeons and the presence of contraindications such as coagulation disorders or thrombocytosis may diminish the feasibility of this approach. Several non-invasive methods are available for diagnosing ICP elevation, including computed tomography (CT) and magnetic resonance imaging (MRI). However, both require patient transportation to imaging facilities, significantly reducing convenience (3).

The optic nerve develops as part of the brain’s protrusion during embryogenesis to form the visual apparatus, with the three layers of meninges extending to form the optic nerve sheath (ONS). The subarachnoid space around the optic nerve communicates directly with the optic chiasm cistern, and this connection allows the cerebrospinal Fluid to move freely in these two contiguous spaces. When ICP changes, the pressure is transmitted via the subarachnoid space to the optic nerve surroundings, leading to an expansion of the optic nerve sheath diameter (ONSD), therefore, ONSD can indirectly reflect ICP levels. Recent studies reported that non-invasive imaging techniques for measuring ONSD offer a novel diagnostic approach for early ICP elevation (2, 4). CT and MRI for ONSD measurement are time-consuming, costly, and inconvenient, therefore, ultrasound (US) assessment of ONSD emerges as a better option due to low cost, rapid bedside performance, and particular suitability for intensive care unit settings and patients requiring real-time ICP monitoring (5). The advantages of US in ICP prediction lie in its accessibility, non-invasiveness, and reproducibility (6, 7). US measurement of ONSD established varying normal values and identified critical ONSD thresholds for assessing ICP elevation (8). However, it remains unclear whether different measurement modalities affect ONSD assessment. Thus, whether US -measured ONSD sensitively evaluate the real-time ICP is unknown.

US measurement of the ONSD is recognized as a safe and effective non-invasive ICP assessment method, particularly suitable for emergency settings and patients unable to undergo direct ICP measurement (9). This non-invasive approach demonstrates high specificity and sensitivity, becoming a focal point of current clinical research. Some experts contend that transverse scanning (ONSLD) at 3 mm behind the globe yields a higher sensitivity in predicting ICP elevation, while others favor the sensitivity of longitudinal scanning (ONSVD). Considering that the optic nerve is encased in a sheath derived from the dura mater, arachnoid mater, and pia mater, forming an elliptical cross-section, some researchers propose that changes in the sheath area may better reflect ICP variations, advocating the use of area measurement as a more convincing approach. In summary, the aim of this study is to find a accurate and non-invasive approach identifying the ICP of patients by US measurement of the ONSD, bringing convenience to clinical practice.

This study explores the ability of different measurement modalities to predict ICP elevation in a cohort of patients with ICP ≥ 200 mmH_2_O at 3 mm behind the globe and validate the use of US-measured ONS for ICP elevation screening and to compare three distinct measurement modalities, aiming to provide a superior non-invasive ICP monitoring technique for clinical practice.

## Methods

### Patients

A total of 104 patients who were scheduled for LP and ICP measurement in the emergency department of neurology at the First Affiliated Hospital of Wenzhou Medical University from January 2018 to May 2021 were enrolled in this study retrospectively. Exclusion criteria were as follows: (1) patients with ocular skin infections, injuries, or a history of intraocular surgery; (2) patients with mental illnesses or agitation that hindered the ocular ultrasonography; (3) failure of ICP measurement via LP due to various reasons. Patients meeting any of the above criteria were excluded from the study. This study was a retrospective analysis and approved by the Ethics Committee of the First Affiliated Hospital of Wenzhou Medical University.

### Data measurement

**Figure 1:**
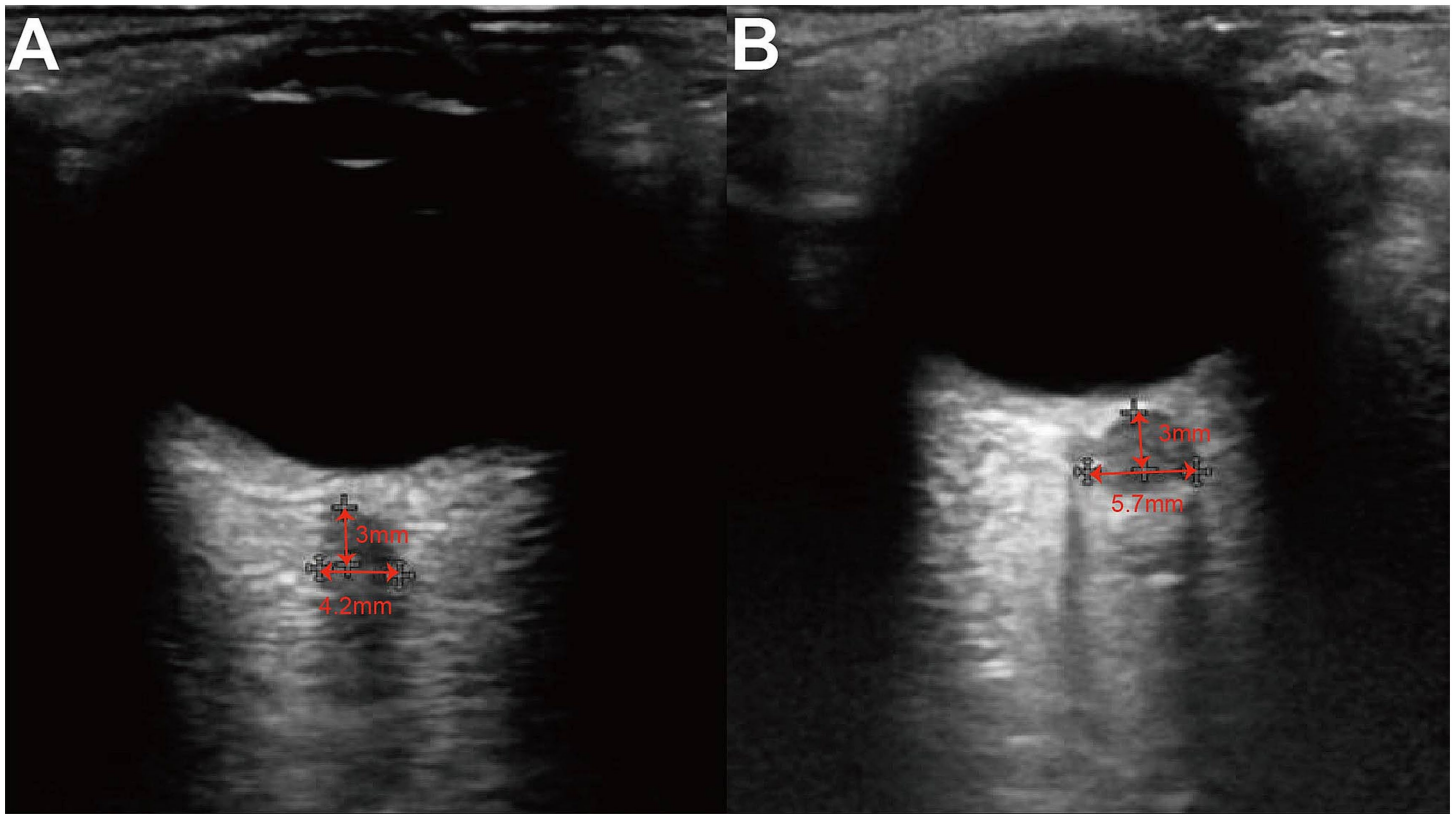
Ultrasound of ONS. The ONSD at 3 mm posterior to the globus was measured from the inner edge of the dura mater to the inner edge of the two vertical hypoechoic lines, by drawing a horizontal line from this point. **(A)** A patient of normal ICP group whose intracranial pressure was 90 mmH_2_O and ONSD was 4.2 mm. **(B)** A patient of high ICP group whose intracranial pressure was 250 mmH_2_O and ONSD was 5.7 mm.

A Hitachi HI VISION Preirus color Doppler ultrasound diagnostic system equipped with an EUP-L74M probe operating at a frequency range of 5–13 MHz was utilized. According to FDA regulations regarding mechanical index and thermal index for ocular US, we used a mechanical index of less than 0.23 and a thermal index of less than 1.0 to avoid damage to the retina and lens. The patients were asked to lie down on the back and close their eyes. The US beam was focused on the retro-orbital region, and gain was adjusted to achieve optimal contrast between the optic nerve and the surrounding fat. The ONSLD and optic nerve sheath vertical diameter (ONSVD) at 3 mm behind the globe was measured bilaterally using both transverse and longitudinal scanning techniques (Figure 1). Horizontal scanning technique refers to placing the probe in a plane perpendicular to the long axis of the optic nerve after clearly displaying the optic nerve sheath structure. Longitudinal scanning technique, on the other hand, means placing the probe in a plane parallel to the long axis of the optic nerve. Assuming the optic nerve to be elliptical, the optic nerve sheath area (ONSA) was calculated as ONSA = ONSLD× ONSVD × *π* / 4, with a precision of 0.01 mm. Based on the baseline IC*p* values obtained from LP, patients were categorized into two groups: (1) Normal ICP group (ICP < 200 mmH_2_O) and (2) High ICP group (ICP ≥ 200 mmH_2_O).

### Statistical analysis

R statistical software (version 4.2.2, https://cloud.r-project.org/) was used for data analysis. The measurement data of abnormal distribution was expressed as median and quartile. Spearman correlation was used to analyze the consistency between all the measured values. Wilcoxon rank-sum test and Chi-squared test were used to compare the differences in age, ONSLD, ONCVD and ONSA between the high ICP and normal ICP groups. The ROC curve was used to obtain the optimal threshold value for diagnosis of high ICP, and the sensitivity, specificity, NPV, PPV and accuracy of the method were evaluated. *p* value <0.05 was considered statistically significant.

## Results

### Clinical characteristic of patients

A total of 104 participants including 70 males (67%) and 34 females (33%) were included in the study, with median age of 48 years (Table 1). The normal ICP group had 52 participants, with a median age of 51 years, while the high ICP group also had 52 participants, with a median age of 42.5 years. There were no statistically significant differences in age (*p* = 0.19) or gender (*p* = 0.4) between the two groups. The measurements in the high ICP group, whether by transverse scan, longitudinal scan, or area method, were all greater than those in the normal IC group, with statistically significant differences between the two groups (*p* < 0.001).

**Table 1:** Clinical characteristic of different ICP groups.

Characteristic	Overall (*N* = 104)	High ICP (*N* = 52)	Normal ICP (*N* = 52)	*P* value
Age	48.00 (33.50,59.00)	42.50 (29.75,59.00)	51.00 (36.50,59.25)	0.190
Gender				0.403
Female	34 (33%)	19 (37%)	15 (29%)	
Male	70 (67%)	33 (63%)	37 (71%)	
ICP	195.00 (145.00,232.50)	235.00 (205.00,276.25)	145.00 (130.00,160.00)	<0.001
Left ONSLD	5.50 (5.18,5.90)	5.90 (5.78,5.93)	5.20 (5.00,5.40)	<0.001
Right ONSLD	5.60 (5.30,5.90)	5.90 (5.70,6.13)	5.30 (5.10,5.50)	<0.001
Left ONSVD	5.65 (5.30,6.00)	6.00 (5.80,6.00)	5.40 (5.08,5.50)	<0.001
Right ONSVD	5.70 (5.40,6.00)	6.00 (5.80,6.30)	5.50 (5.30,5.70)	<0.001
Left ONSA	24.40 (21.50,27.32)	27.32 (26.40,28.25)	21.63 (19.92,22.99)	<0.001
Right ONSA	25.06 (22.47,27.78)	27.77 (25.95,30.29)	22.65 (21.11,24.29)	<0.001

### The correlation between clinical characteristics

**Figure 2:**
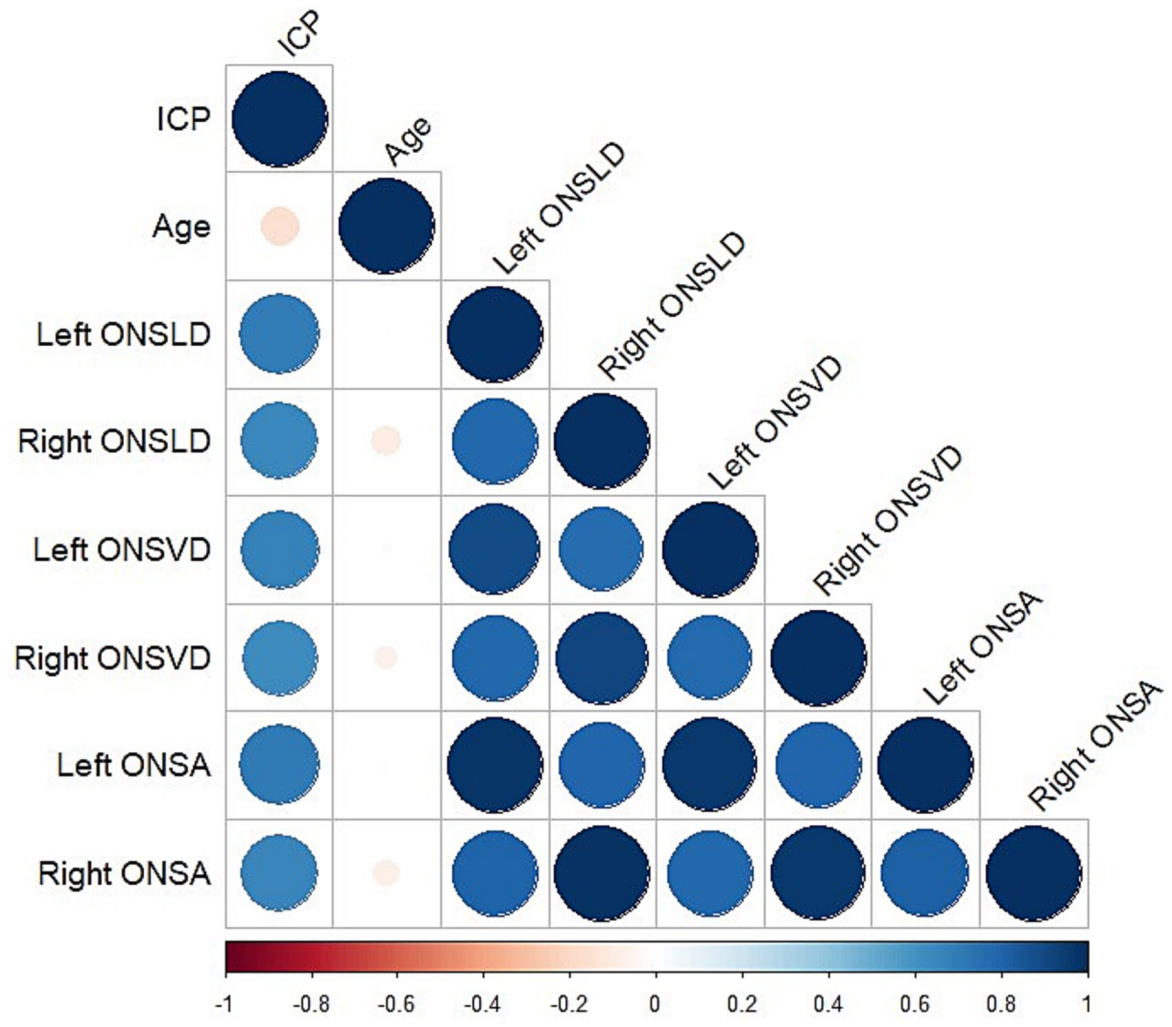
Heatmap of correlation between all characteristics. Color and size of each circle indicated the degree of correlation. Blue represented positive correlation; red represented negative correlation.

Spearman correlation analysis was used between clinical characteristics (Figure 2). Significant correlations were observed between ICP and the left ONSLD (*r* = 0.69, *p* < 0.001), the right ONSLD (*r* = 0.65, *p* < 0.001), the left ONSVD (*r* = 0.68, *p* < 0.001), the right ONSVD (*r* = 0.62, *p* < 0.001), the left ONSA (*r* = 0.71, *p* < 0.001) and the right ONSA (*r* = 0.65, *p* < 0.001). Conversely, no significant correlations were observed between ICP and age (*r* = −0.16, *p* = 0.11).

### The efficacy of ONSLD, ONSVD and ONSA in diagnosing ICP

**Figure 3:**
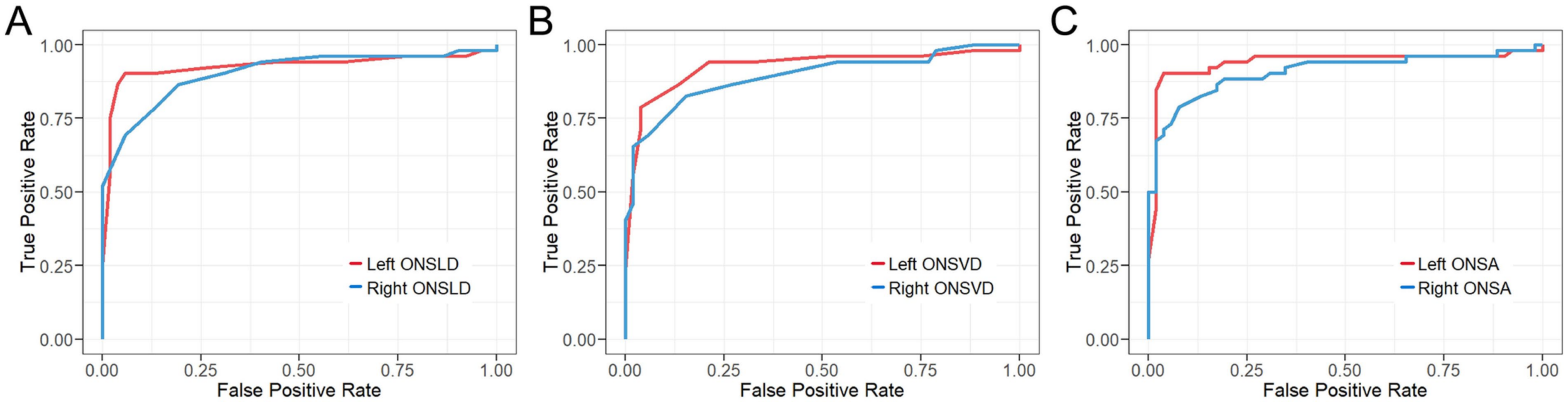
ROC curves of **(A)** ONSLD, **(B)** ONSVD and **(C)** ONSA predicting ICP.

ROC curve analysis determined that the cutoff value for diagnosing ICP (Figure 3 and Tables 2, 3). The cutoff value of left ONSLD is 5.55 mm (AUC = 0.93, 95% CI = 0.87–0.99), with a diagnostic sensitivity of 90.38%, specificity of 94.23%, PPV of 94%, NPV of 90.74% and accuracy of 92.31%. The cutoff value of right ONSLD is 5.55 mm (AUC = 0.91, 95% CI = 0.84–0.96), with a diagnostic sensitivity of 86.54%, specificity of 80.77%, PPV of 81.82%, NPV of 85.71% and accuracy of 83.65. The cutoff value of left ONSVD is 5.75 mm (AUC = 0.93, 95% CI = 0.87–0.98), with a diagnostic sensitivity of 78.85%, specificity of 96.51%, PPV of 95.35%, NPV of 81.97% and accuracy of 87.5%. The cutoff value of right ONSVD is 5.75 mm (AUC = 0.89, 95% CI = 0.83–0.96), with a diagnostic sensitivity of 83.69%, specificity of 84.62%, PPV of 84.31%, NPV of 83.12% and accuracy of 83.65%. The cutoff value of left ONSA is 25.04 mm^2^ (AUC = 0.94, 95% CI = 0.88–0.99), with a diagnostic sensitivity of 90.38%, specificity of 96.15%, PPV of 95.92%, NPV of 90.91% and accuracy of 93.27%. The cutoff value of right ONSA is 25.73 mm^2^ (AUC = 0.91, 95% CI = 0.85–0.97), with a diagnostic sensitivity of 78.85%, specificity of 92.31%, PPV of 91.11%, NPV of 81.36% and accuracy of 85.58%. According to the above analysis, if ONSLD >5.55 mm, ONSVD >5.75 mm, left ONSA >25.05 mm^2^ or right ONSA >25.73 mm^2^, the patient was considered to have elevated ICP.

**Table 2:** AUC and CI of predictors.

Predictor	Left	Right
ONSLD, AUC (95% CI)	0.9268 (0.8655,0.988)	0.9044 (0.8432,0.9656)
ONSVD, AUC (95% CI)	0.9262 (0.8699,0.9829)	0.8948 (0.8317,0.9579)
ONSA, AUC (95% CI)	0.9406 (0.8847,0.9966)	0.9087 (0.847,0.9703)

**Table 3:** Best cut-off and effectivity of predictors.

Predictor	Cut-off	Sensitivity	Specificity	PPV	NPV	Accuracy
Left ONSLD	5.55	90.38%	94.23%	94%	90.74%	92.31%
Right ONSLD	5.55	86.54%	80.77%	81.82%	85.71%	83.65%
Left ONSVD	5.75	78.85%	96.15%	95.35%	81.97%	87.5%
Right ONSVD	5.75	83.69%	84.62%	84.31%	83.12%	83.65%
Left ONSA	25.04	90.38%	96.15%	95.92%	90.91%	93.27%
Right ONSA	25.73	78.85%	92.31%	91.11%	81.36%	85.58%

## Discussion

Elevated ICP can be induced by various cerebral disorders, such as stroke, hemorrhage, malignancies, and trauma (10). Timely diagnosis and treatment of increased ICP can prevent dangerous outcomes like cerebral hernia and reduce mortality rates (11). Although invasive ICP monitoring is considered the “gold standard,” it requires high clinical expertise and may be associated with postoperative complications, including infection and hemorrhage (12). Signs of raised ICP manifest as ventricular obliteration and midline shift on CT and MRI scans, yet these imaging modalities have their limitations (13). Over the past two decades, studies suggest that bedside US measurement of ONSD serve as an alternative to ICP monitoring. In ICU, outpatient clinics, and emergency departments, widened ONSD has been observed in patients with ICP elevation due to various etiologies (14, 15). Recently, optic nerve ultrasonography has been validated as a noninvasive, accurate, safe, repeatable, and cost-effective tool for ICP monitoring through ONSD measurements, thereby mitigating the potentially harmful consequences of invasive ICP measurements. Despite years of research, consensus has yet to be reached on the threshold ONSD value indicative of elevated ICP. Though not a novel concept, with over two decades since its initial investigation, the limited number of published clinical studies may be attributed to US not being a commonly employed diagnostic tool in the past few years (16, 17). This study aims to investigate the correlation between different measurement methods of the retrobulbar ONS and ICP, as well as the cutoff values, sensitivity, and specificity for diagnosing ICP elevation.

Our study, examining the relationship between the characteristics of retrobulbar ONS and ICP in 104 patients, found that US measurement of the retrobulbar ONS may be a robust predictor of ICP elevation, demonstrating high sensitivity and specificity. Prior studies have already established the reliability of ONSD measurements in assessing ICP, and our findings were consistent with their findings. While invasive ICP monitoring remains the gold standard, its routine use is limited in many hospitals due to unavailability of monitoring devices, contraindications, and high costs. This study provides additional evidence supporting the rationale for real-time ICP monitoring using US-based ONSD measurements.

Initially, before the widespread adoption of US, non-invasive methods including CT and MRI were used to diagnose elevated ICP, both of which exhibited high sensitivity (18, 19). With the increasing popularity of US, the unique convenience and reproducibility have garnered significant attention for US-based ICP diagnosis (20). Li et al. published a study in 2018, focusing on the correlation between US-measured ONSD and ICP, as well as its feasibility in predicting ICP elevation. Their study involved 130 patients scheduled for ICP measurement via LP in neurology outpatient clinics. Similar to our results, they found an optimal cutoff value of >5.6 mm, with a sensitivity of 86% and a specificity of 71%. In our study, both transverse and longitudinal scanning demonstrated similar sensitivities to Li′s, but with higher specificity, which could be attributed to differences in US equipment and operator error. Subsequently, we focused on the correlation between ONSA measurements and ICP, and the ability to predict ICP elevation. ROC curve analysis revealed comparable areas under the curve for all three measurements.

To date, researchers continue to use US to measure changes in ONSD as a proxy for ONS volume changes. However, it must be acknowledged that the ONS may not be a perfectly circular structure. Killer et al. studied 12 optic nerves in 9 patients and found that the subarachnoid space around the human optic nerve is not a uniform, cerebrospinal fluid-filled cavity (21). Instead, it contains a complex system of arachnoid trabeculae and septations dividing the subarachnoid space, with varying structures and volumes depending on the location within the optic nerve. Consequently, ONSD measurements can vary significantly between patients and within different locations of the sheath. To enhance measurement accuracy, we performed both transverse and vertical scans, and the average represented the change in ONSD in this study. For cases where ONSD changes do not adequately reflect ONS volume changes, area measurements may be crucial, which is why the ONSA was studied.

A recent meta-analysis aimed to evaluate the ability of US-measured ONSD as a non-invasive indicator of ICP. This review encompassed seven prospective studies including 320 patients published over 27 years from 1990, all of which involved invasive ICP monitoring as the gold standard for diagnosing high ICP, excluding studies relying solely on radiological signs to minimize heterogeneity. All included studies used a threshold of at least 20 mmHg to diagnose high ICP. Among the seven studies, the cutoff values for US-measured ONSD ranged from 4.8 mm to 6.3 mm. The sensitivity of US-measured ONSD ranged from 88 to 94% in diagnosing high ICP compared to invasive ICP measurements. The high sensitivity and relatively low specificity make US-based ONSD measurements an excellent screening tool for bedside diagnosis of high ICP (13).

Another recent meta-analysis aimed to determine the optimal ONSD threshold for identifying elevated ICP, which remains an unresolved issue. A systematic literature review of online databases from 2003 to 2020 included 22 studies with 779 patients. The mean ONSD in patients with ICP elevation was >5.82 mm (95% CI = 5.58–6.06). Variations in ONSD were observed based on the etiology of ICP, clinical settings for ONSD measurement, and criteria for diagnosing ICP. Our results fell within this range. Though a definitive threshold remains elusive, our work contributes to future studies assessing the sensitivity and specificity of US-based ONSD measurements in diagnosing ICP. Meta-analyses represent the most up-to-date and reliable evidence for using US to diagnose high ICP. However, from our perspective, the most significant limitation lies in the heterogeneity of patient diagnoses across studies. We believe this represents a strength of our study, as we exclusively selected patients with elevated ICP.

Our study also has limitations. Firstly, the sample size is relatively small, necessitating future studies with larger cohorts. Secondly, patients in this study were from neurology departments and did not include those with extremely high ICP; future studies should increase the sample size of critically ill patients to enhance insights into this population. And, combining other non-invasive methods with US for ICP assessment merits consideration in future research. The use of B-scan technology in assessing ONSD may causes a risk of error due to the blooming effect which consists of variations in the measured size of the structure and depends on sensitivity settings (22). But standardized A-scan technology is not affected by the blooming effect and can yield more accurate ONSD measurements due to the distinct high-reflection arachnoid spike (23). However, in this study, due to instrumental limitations and the lack of specialized training in relevant A-scan techniques, we utilized B-scan technology. Additionally, to reduce the errors in US-measurements, every measurements were completed by two doctors with more than five years of work experience. Finally, individual variations in the optic nerve should be considered in future studies.

## Conclusion

Ultrasonic transverse scanning, longitudinal scanning, and area-based measurement of the retrobulbar ONS are excellent screening tools for the diagnosis of ICP. These three methods exhibited nearly identical levels of correlation, sensitivity, and specificity. All three measurement approaches demonstrated capabilities in diagnosing elevated ICP.

## Strengths and limitations of this study

1. Optic nerve sheath is a noninvasive diagnostic parameter.
2. The sample size is relatively small.
3. Patients in this study did not include those with extremely high ICP.

## Data Availability

The raw data supporting the conclusions of this article will be made available by the authors, without undue reservation.
